# An unexpected insight into the cause of olfactory dysfunction: fibrillogenesis of odorant-binding proteins

**DOI:** 10.1038/s41420-025-02671-x

**Published:** 2025-08-07

**Authors:** Olga V. Stepanenko, Maksim I. Sulatsky, Anna I. Sulatskaya, Olesya V. Stepanenko

**Affiliations:** 1 Laboratory of Structural Dynamics, Stability and Folding of Proteins, Institute of Cytology, Russian Academy of Sciences, St. Petersburg, Russia; 2 Laboratory of Cell Morphology, Institute of Cytology, Russian Academy of Sciences, St. Petersburg, Russia

**Keywords:** Protein aggregation, Cell death in the nervous system

## Abstract

Olfactory dysfunction is a common complication of serious pathologies, including neurodegenerative disorders, bacterial and viral infections, including COVID-19, and others. Despite the widespread prevalence of olfactory disorders, the pathophysiological mechanisms of their development, as well as the molecular basis of their association with the underlying disease, remain incompletely understood. The current work formulates a new concept of the origin of olfactory disorders, linking a decrease in the activation of olfactory neurons and their death to the fibrillogenesis of odorant-binding proteins (OBPs), which are the primary participants of olfactory perception. The potential triggers of OBPs’ amyloidogenesis in vivo are discussed, such as molecular crowding, components of nasal medications, environmental factors, and cross-seeding with viral and bacterial amyloids. Several ways of impairment of olfactory signaling as a result of fibrillogenesis of OBPs are formulated: complete loss of OBPs functionality following amyloid formation; mechanical blockage of the membranes of sensory neurons and damage to chemoreceptors on their surface, preventing olfactory signaling; cytotoxic effect of OBPs’ amyloid on sensory neurons and other cells of the olfactory epithelium. The proposed concept offers a novel perspective on the pathogenesis of olfactory dysfunction, as well as its possible association with amyloidoses, including in neurodegenerations, and infectious diseases. It opens prospects for the development of new therapeutic approaches to the treatment of olfactory disorders.

## Introduction

The sense of smell is one of the five basic human senses. It indicates physical health, ensures social adaptation, and helps in avoiding life- and health-threatening situations. Meanwhile, the complete loss of the sense of smell (anosmia) or a marked loss of ability to discriminate smells, temporary or chronic, is a widespread pathological condition [1]. In most cases, olfactory impairment is not an independent disease, but rather a concomitant complication of various pathologies, including neurodegenerative disorders [2]. Despite the widespread prevalence of anosmia and hyposmia, the pathophysiological mechanisms of their development, as well as the molecular basis of their association with the underlying disease, remain incompletely understood. Although several molecular mechanisms contributing to olfactory dysfunction have been identified, the underlying cause remains unknown in approximately 20% of cases, which are therefore classified as idiopathic [3, 4]. In this context, we propose a novel concept linking olfactory dysfunction to the fibrillogenesis of odorant-binding proteins (OBPs), which impairs the activation of olfactory neurons and disrupts odor perception. In this context, we propose a novel concept linking olfactory dysfunction to the fibrillogenesis of OBPs, involved in odor perception, which impairs the activation of olfactory neurons. The concept provides a new perspective on the pathogenesis of olfactory dysfunction and reveals its possible association with the development of amyloidoses, particularly those associated with neurodegenerative diseases. It also offers prospects for developing new therapeutic approaches to treat olfactory disorders.

## Olfactory dysfunction and related pathologies

Olfactory disorders are observed across a broad spectrum of conditions, with considerable variation in their underlying causes and clinical manifestations [2, 4]. This pathology can be observed both in hereditary anomalies (Sievert-Kartagener and Kallmann syndromes) and following upper respiratory tract infections, including acute respiratory viral infections, sinusitis, and COVID-19. Olfactory dysfunction can also be caused by mechanical damage to the paranasal sinuses or exposure to narcotic and toxic substances (pesticides, ammonia), resulting in disorders of varying severity and duration. A serious risk of olfactory loss is also associated with neurological damage in diseases such as meningoencephalitis, traumatic brain injury and benign and malignant tumors of the central nervous system (CNS). Olfactory impairment is also a common feature of neurodegenerative disorders, such as Parkinson’s and Alzheimer’s diseases. A gradual decline in olfactory function is also commonly observed as part of the natural aging process. Obviously, the variety of diseases associated with smell perception disorders indicates the multifactorial nature of the underlying causes.

## The established causes of olfactory dysfunction

The transmission of odor stimuli begins with the activation of G-protein-coupled receptors in the membrane of olfactory sensory neurons of the olfactory epithelium by the action of odorant molecules [5]. The subsequent triggering of signaling cascades ensures the nerve impulse transmission by receptor neurons to the olfactory bulbs and then by mitral and tufted neurons to the brain centers responsible for the formation of the olfactory image. Given this, current thinking suggests the following causes for developing olfactory dysfunction [6]: (1) impaired functioning of peripheral olfactory structures (namely, reduced intensity of primary activation of olfactory receptor neurons) through mechanical blockage of odorant access or damaging the nasal mucosa; (2) lesions of the sensory olfactory system, including olfactory tract transmitter neurons and CNS structures (piriform cortex, amygdala, olfactory tubercle and parahippocampal gyrus). There are some reported cases of olfactory dysfunction resulting from mutations in the genes of molecular participants in olfactory perception and signal transduction (signaling cascade proteins and ion channels of neuronal synapses) [7, 8]. At the same time, known pathological mechanisms explain most, but hardly all, cases of olfactory dysfunction.

## Focus on OBPs

We observed that impaired functioning of transporter proteins represented by OBPs in nasal mucus has not been considered a possible cause of anosmia/hyposmia until now. Odorant molecules, mostly water-soluble, have to pass through a layer of hydrophilic mucus to interact with olfactory neuron receptors. These perireceptor events involve OBPs, which reversibly bind and thus solubilize odorant molecules [9, 10].

The ability to bind odorant molecules originates from the specific structure of OBPs. They are small secretory proteins with a molecular mass of about 18 kDa. OBPs’ polypeptide chain is folded into a beta-barrel of 9 antiparallel beta-strands flanked by C-terminal alpha-helical segments [9]. Precisely in the beta-barrel cavity, hydrophobic odorant molecules are captured. Thus, the integrity of the unique spatial organization of OBPs is clearly crucial to maintaining their function and is required for these proteins to provide the primary steps of olfactory perception.

## Fibrillogenesis of OBPs in vitro

Reversible OBPs’ denaturation has probably no significant effect on their functional activity. Complete loss of function requires a mechanism leading to an irreversible change in the OBPs’ spatial structure. Our recent work series has demonstrated the ability of proteins of this class to form amyloid fibrils, ordered protein aggregates enriched with cross-beta structure [11–14]. The structural transformation of OBPs’ beta-barrel during fibrillogenesis has been shown to disrupt the functional core of these proteins [11]. Fibrillogenesis of OBPs is initiated by subtle changes in their native structure, stabilized by the disulfide bonding of the protein C-terminal domain to the beta-barrel [14]. It has been shown that disorganization of regions of ordered structure in the C-terminal part of the protein, including a small alpha-helical segment and a short beta9-strand enclosing the beta-barrel, leads to weakening of intramolecular native contacts. The emerging accessible ‘sticky’ amyloidogenic sites on the protein surface promote intermolecular interactions and subsequent fibrillogenesis. The oligomerization of OBPs has been identified as a key event in the early stages of fibrillogenesis, triggering all subsequent structural transformations of the protein scaffold [12]. Specifically, oligomerization initiates a cascade of gradual changes in the weakened beta-barrel structure. This process begins with the disruption of one of the beta-strands, followed by the opening of the protein core. Ultimately, these changes culminate in a global reorganization of the barrel’s beta-sheet, as the beta-strands align into a common register, resulting in the formation of mature amyloid fibrils. It turned out that the loss of ligand-binding activity and the appearance of pronounced cytotoxic properties inherent in mature amyloid OBPs are manifested already at the formation of prefibrillar aggregates with a significantly reorganized beta-barrel [13].

## Potential initiating factors of OBPs’ fibrillogenesis in vivo

**Fig. 1:**
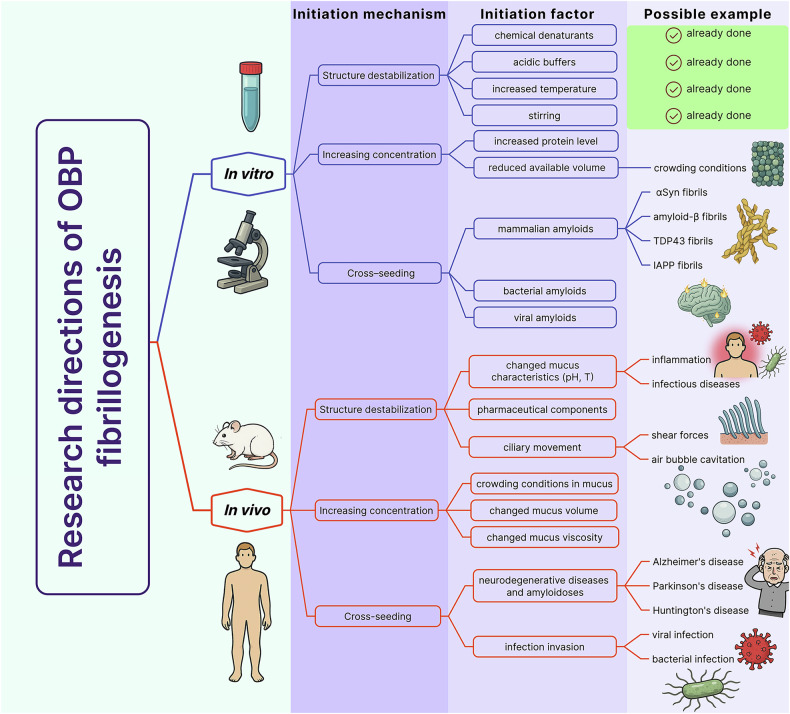
Proposed research directions of OBP fibrillogenesis. A conceptual framework outlining potential pathways that may initiate fibrillogenesis of odorant-binding proteins (OBPs) under in vitro and in vivo conditions. The diagram highlights key underlying mechanisms that trigger OBP fibrillogenesis – including structural destabilization, increased local concentration, and cross-seeding with other amyloids – and its possible initiation factors. While some in vitro conditions have already been explored (indicated with checkmarks), in vivo scenarios remain unstudied and represent prospective directions for future investigation into the pathological relevance of OBP aggregation. Created with Figma and Adobe Photoshop.

Structural changes that weaken the OBPs’ beta-barrel and initiate fibrillogenesis cascade can be triggered by mild destabilizing stresses, even under physiological temperature and pH conditions (Fig. 1). Based on our in vitro findings, we hypothesize that OBPs’ fibrillogenesis may also occur in vivo as a result of single or cumulative exposure to the factors outlined below.

1. Olfactory mucus that harbors OBPs in vivo contains many components: mucoglycoproteins that determine the viscous properties of mucus, immunoglobulins, cytokines, many enzymes (including lysozyme, which exhibits antibacterial activity, and enzymes involved in detoxification), and salts. Given that oversaturated conditions, known as molecular crowding conditions, play an important role in accelerating the fibrillogenesis of various amyloidogenic proteins and peptides (by increasing their local concentration) [15, 16], there is a strong possibility that such an environment may also promote the aggregation of OBPs.
2. A reduction in nasal secretion volume or an increase in its viscosity—such as during dehydration or chronic inflammation of the upper respiratory tract [17, 18]—may not only elevate the local concentration of OBPs, which are normally present at relatively high millimolar levels [9]. In addition, changes in mucus properties may directly interfere with the trafficking of odorants by OBPs to the sensory neurons of the olfactory epithelium and affect the activation of receptors on their surface. These conditions may also facilitate their ordered aggregation, both because of increased local concentration of the protein and as a result of crowding effects.
3. External factors destabilizing the OBPs’ structure can also facilitate the formation of amyloid fibrils by these proteins. The ciliary movement in the mucus layer can already cause such effects on proteins as a result of shear forces and/or cavitation of air bubbles formed in this process and a number of other effects [19, 20].
4. The destabilizing effects can be enhanced by changes in the characteristics of olfactory mucus. For example, OBPs were found to form amyloid structures more efficiently in vitro with decreasing medium acidity and increasing temperature. In vivo, similar changes in mucus properties have been observed in various inflammatory processes (including rhinosinusitis) and in infectious diseases [21].
5. Fibrillogenesis-promoting disturbance of OBPs’ structure can be caused by pharmaceutical components administered through the nose for local or systemic action. For example, a number of nasal sprays with anti-inflammatory, antibacterial, and mucolytic effects can lead to the disruption of native disulfide bonds in OBPs, causing their destabilization. For example, such an effect may be exerted by acetylcysteine, an effective reducing agent contained in high acting concentrations in mucolytic preparations [22].
6. Another trigger of OBPs’ fibrillogenesis may be cross-seeding initiated by amyloid of different origins. In particular, some observations point to the occurrence of olfactory dysfunction in a number of neurodegenerative diseases (Alzheimer’s, Parkinson’s, Huntington’s, frontotemporal dementia) [23, 24]. Taking into account these observations and the demonstrated by us propensity of OBPs to form fibrils under the action of factors of different nature, it can be assumed that their fibrillogenesis can be induced, among others, by the amyloid seed accumulated in neurodegenerative diseases. According to the literature, such cross-interactions between amyloidogenic proteins/peptides (e.g., beta-amyloid with alpha-synuclein, islet amyloid polypeptide or TDP-43) may explain the overlapping symptomatic manifestations in different neurodegenerative diseases and amyloidoses [25–29]. In addition, some studies discuss the possibility of systemic circulation of amyloid aggregates between the brain and peripheral tissues [30]. In particular, it is hypothesized that pathologies associated with alpha-synuclein aggregation may propagate through the nasal-brain axis [31]. It should be noted that cross-seeds can have not only endogenous but also exogenous origin.

## Exogenous seeds as candidate triggers for OBPs’ fibrillogenesis in vivo

As nasal mucus has barrier and protective functions, it traps pathogenic bacterial and viral particles, preventing them from reaching the olfactory epithelium and minimizing the risk of infection. In individuals with olfactory dysfunction, the composition of the nasal mucus microbiome changes towards the predominance of pathogenic bacterial strains [32]. Growing evidence suggests that pathogenic microorganisms can exploit proteins in the amyloid state in their life cycle [33, 34]. A well-known example is biofilm formation by pathogenic bacteria with the participation of extracellular fibrillar structures known as curli. Furthermore, specific features of viral evolution and replication cycles are thought to underlie the high amyloidogenic potential of many viral proteins [35]. There are increasing indications in the literature of cross-interactions between mammalian proteins and virus/bacterial proteins. Specifically, the formation of amyloid fibrils of the SARS-CoV-2 spike protein is thought to be associated with the conversion of fibrinogen to the amyloid form during abnormal blood clotting, one of the severe complications of viral infection [36]. Cross-seeding between amyloidogenic viral and host proteins is considered among several mechanisms [35, 37] contributing to amyloid propagation and explaining the increased risk following viral infections of amyloidoses, including in neurodegenerative diseases [38]. Induced aggregation of α-synuclein, whose fibrils are formed in various synucleinopathies, is observed in the presence of amyloids of cell surface curli of gut bacteria [39]. It has also been suggested that gut microbiota dysbiosis may be involved in the development of Alzheimer’s and Parkinson’s disease through several mechanisms, including curli-dependent induction of amyloid-beta protein aggregation, which may begin beyond the brain in the gastrointestinal tract [30, 40–42]. Taking into account these data, we can highlight another mechanism of cross-induction of OBPs’ fibrillogenesis associated with their interaction with amyloid fibrils of viruses and pathogenic bacteria, similar to the mechanism proposed earlier for endogenous amyloid seeds.

Thus, many factors can initiate fibrillogenesis of OBPs in vivo. Next, we consider the consequences of such irreversible transformations of OBPs in the body.

## Possible effects of OBPs fibrillogenesis on olfactory receptor neuron viability and functioning

**Fig. 2:**
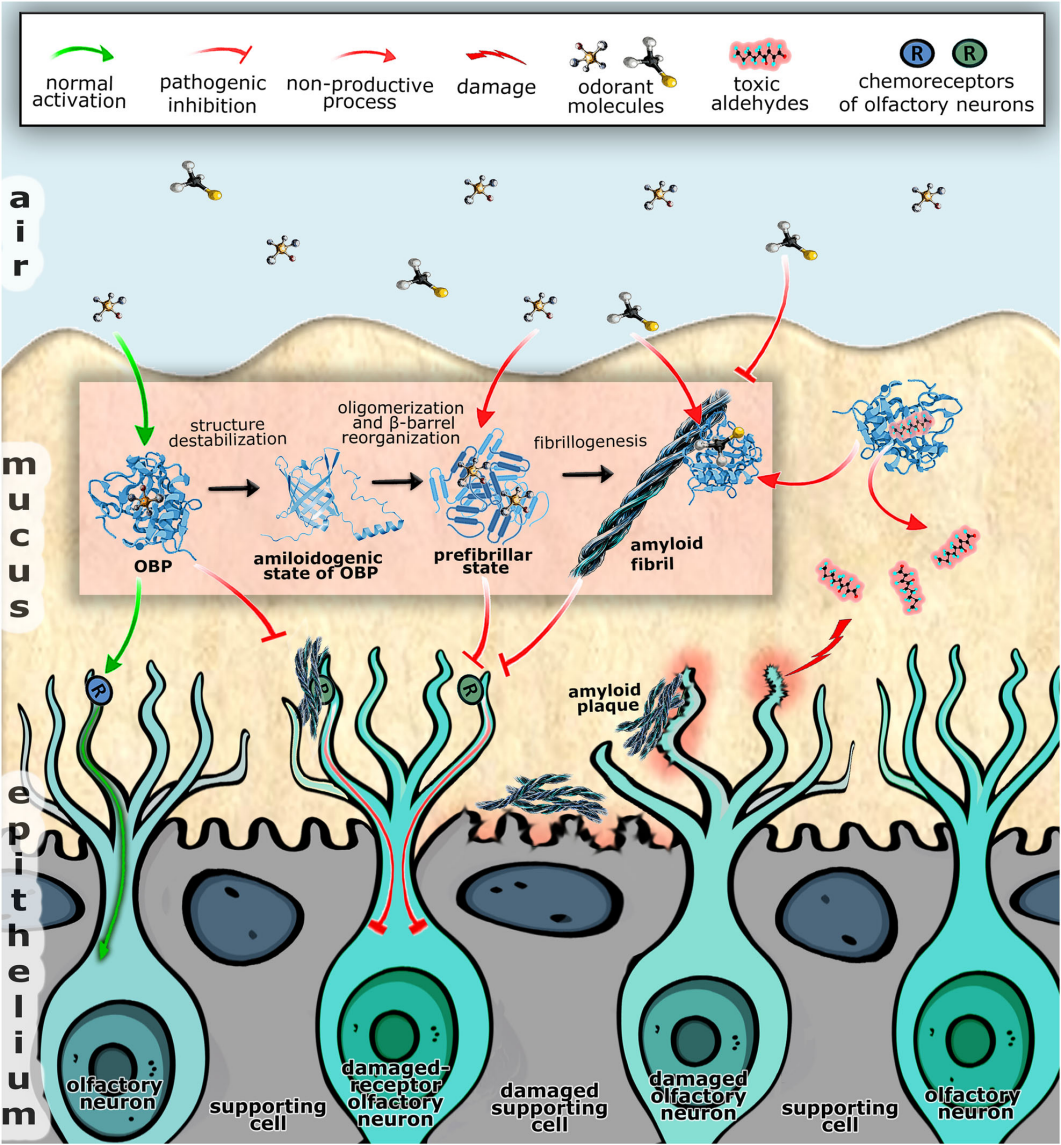
Potential outcomes of OBPs’ fibrillogenesis. Fibrillogenesis of OBPs is initiated by subtle changes in their native structure, generating an amyloidogenic monomeric protein state. Oligomerization of protein molecules in this amyloidogenic state through intermolecular interactions of amyloidogenic regions leads to a series of beta-barrel transformations with the formation of prefibrillar and fibrillar states. These events, illustrated schematically in the figure, may disrupt normal olfactory signaling (shown by green arrows on the left side). Several mechanisms of interference by OBPs’ fibrillogenesis with the signaling cascade are proposed: loss of OBPs functionality following formation of amyloid, as well as their prefibrillar state (shown by red arrows in the middle); mechanical blockage of the membranes of sensory neurons and damage to chemoreceptors on their surface, preventing olfactory signaling (shown by red T-shaped arrow on the left side); cytotoxic effect of OBPs’ amyloid on sensory neurons and other cells of the olfactory epithelium (indicated at the contact sites with fibrillar plaques in the middle). Fibrillar aggregates of OBPs can further incorporate and initiate the conversion of functionally active OBPs into fibrillar form (shown by standard red arrows), depleting their pool, thereby interfering with olfactory receptor functioning and enhancing oxidative stress (shown by a lightning-shaped arrow on the right side). Created with Figma and Adobe Photoshop.

Given the functional role of native OBPs and the change in their properties upon ordered aggregation, we hypothesize that fibrillogenesis of OBPs may disrupt the early steps of olfactory signaling. This process could be implemented through several mechanisms (Fig. 2).

First, because of the functional activity loss during the transition to the fibrillar (and prefibrillar) state, OBPs molecules can no longer participate in delivering odorants to olfactory receptors, rendering their activation impossible.

Moreover, even the formation of a few fibrils of OBPs can lead to further avalanche growth of their amyloid. These primary aggregates may serve as seeds to initiate the transition of increasing numbers of functionally active OBPs monomers into the fibrillar form, depleting the pool of available odorant transporters.

Second, fibrillar and prefibrillar structures, as well as native monomeric OBPs, can build up large clumps that can accumulate on the receptor neuron membranes, creating a physical barrier to the normal interaction of odorant molecules with chemoreceptors. Moreover, incorporating native monomeric proteins into such assemblies will further deplete the active transporter fraction in mucus.

The third consequence of OBPs’ fibrillogenesis may be damaging to membrane-integrated chemoreceptors. Several studies imply that OPBs can scavenge toxic aldehydes from lipid oxidation [43]. This means that impaired functionality of OPBs may contribute to oxidative stress.

Finally, the high cytotoxicity of amyloid and prefibrillar aggregates of OBPs may lead to degeneration of receptor neurons and olfactory epithelial cells that maintain mucosal structure.

## Perspective for further research

This work proposes the concept that OBPs’ fibrillogenesis may be one of the currently missing factors explaining the mechanisms of olfactory dysfunction, including of idiopathic nature. In the context of this hypothesis, we consider several possible pathways of the pathological process: complete loss of OBPs functionality following amyloid formation; mechanical blockage of the membranes of sensory neurons and damage to chemoreceptors on their surface, preventing olfactory signaling; cytotoxic effect of OBPs’ amyloid on sensory neurons and other cells of the olfactory epithelium. Verifying this hypothesis and elucidating the mechanisms of OBP-induced olfactory dysfunction will require further investigation into several key aspects (Fig. 1). First, confirmation of the formation of OBPs’ amyloid in animal models and in human clinical samples (for example, analysis of nasal mucus probes or histologic preparations by electron microscopy or immunohistochemistry) is essential. Such studies should focus on conditions involving environmental extremes, exposure to medications, infectious agents, and neurodegenerative diseases. This will help to determine the true origins of ordered OBPs’ aggregation in vivo. With confirmation of our concept, the next stage of research will involve identifying possible links between OBPs’ fibrillogenesis and other pathological conditions. In particular, analyzing the cross-fibrillogenesis of OBPs with amyloidogenic proteins/peptides from humans, as well as from viruses and pathogenic bacteria, will help to establish the pathways of the amyloid cascade in olfactory dysfunction in the context of infectious and neurodegenerative diseases. The results of the proposed studies could provide the basis for developing therapeutic strategies aimed at stabilizing the OBPs’ structure and inhibiting their aggregation. This, in turn, may open new perspectives for the treatment of olfactory dysfunction.

## References

[1] Boesveldt S, Postma EM, Boak D, Welge-Luessen A, Schopf V, Mainland JD, et al. Anosmia-A clinical review. Chem. Senses. 2017;42:513–23. 28531300 10.1093/chemse/bjx025 PMC5863566

[2] Whitcroft KL, Altundag A, Balungwe P, Boscolo-Rizzo P, Douglas R, Enecilla MLB, et al. Position paper on olfactory dysfunction: 2023. Rhinology. 2023;61:1–108. 37454287 10.4193/Rhin22.483

[3] Keller A, Malaspina D. Hidden consequences of olfactory dysfunction: a patient report series. BMC Ear, Nose Throat Disord. 2013;13:8. 23875929 10.1186/1472-6815-13-8 PMC3733708

[4] Schafer L, Schriever VA, Croy I. Human olfactory dysfunction: causes and consequences. Cell Tissue Res. 2021;383:569–79. 33496882 10.1007/s00441-020-03381-9 PMC7835667

[5] Munger SD, Leinders-Zufall T, Zufall F. Subsystem organization of the mammalian sense of smell. Annu. Rev. Physiol. 2009;71:115–40. 18808328 10.1146/annurev.physiol.70.113006.100608

[6] Guekht AB, Kryukov AI, Kazakova AA, Akzhigitov RG, Gulyaeva NV, Druzhkova TA. Disorders of olfaction – an interdisciplinary problem. Neurosci. Behav. Phys. 2023;53:966–72.

[7] McEwen DP, Koenekoop RK, Khanna H, Jenkins PM, Lopez I, Swaroop A, et al. Hypomorphic CEP290/NPHP6 mutations result in anosmia caused by the selective loss of G proteins in cilia of olfactory sensory neurons. Proc. Natl. Acad. Sci. USA. 2007;104:15917–22. 17898177 10.1073/pnas.0704140104 PMC2000398

[8] Kingwell K. Nav1.7 withholds its pain potential. Nat. Rev. Drug Discov. 2019;18:321–3. 10.1038/d41573-019-00065-0 31048807

[9] Heydel JM, Coelho A, Thiebaud N, Legendre A, Le Bon AM, Faure P, et al. Odorant-binding proteins and xenobiotic metabolizing enzymes: implications in olfactory perireceptor events. Anat. Rec. 2013;296:1333–45. 10.1002/ar.22735 23907783

[10] Boichot V, Muradova M, Nivet C, Proskura A, Heydel J-M, Canivenc-Lavier M-C, et al. The role of perireceptor events in flavor perception. Front Food Sci. Technol. 2022;2:989291.

[11] Stepanenko OV, Sulatskaya AI, Sulatsky MI, Mikhailova EV, Kuznetsova IM, Turoverov KK, et al. Mammalian odorant-binding proteins are prone to form amorphous aggregates and amyloid fibrils. Int J. Biol. Macromol. 2023;253:126872. 37722633 10.1016/j.ijbiomac.2023.126872

[12] Stepanenko OV, Sulatsky MI, Mikhailova EV, Rychkov GN, Sulatskaya AI, Stepanenko OV. Comprehensive picture of beta-barrel transformation in the fibrillogenesis of odorant-binding proteins. Int J. Biol. Macromol. 2025;309:142709. 40174819 10.1016/j.ijbiomac.2025.142709

[13] Stepanenko OV, Sulatsky MI, Sulatskaya AI, Mikhailova EV, Stepanenko OV. Characterization of intermediate states in the fibrillogenesis of odorant-binding proteins. Int J. Biol. Macromol. 2024;282:137412. 39521223 10.1016/j.ijbiomac.2024.137412

[14] Sulatskaya AI, Stepanenko OV, Sulatsky MI, Mikhailova EV, Kuznetsova IM, Turoverov KK, et al. Structural determinants of odorant-binding proteins affecting their ability to form amyloid fibrils. Int J. Biol. Macromol. 2024;264:130699. 38460650 10.1016/j.ijbiomac.2024.130699

[15] Torres-Bugeau CM, Borsarelli CD, Minahk CJ, Chehin RN. The key role of membranes in amyloid formation from a biophysical perspective. Curr. Protein Pept. Sci. 2011;12:166–80. 21348838 10.2174/138920311795860197

[16] Menon S, Sengupta N. Influence of crowding and surfaces on protein amyloidogenesis: A thermo-kinetic perspective. Biochim. Biophys. Acta Proteins Proteom. 2019;1867:941–53. 30928692 10.1016/j.bbapap.2019.03.009

[17] Shinogi J, Harada T, Nonoyama T, Kishioka C, Sakakura Y, Majima Y. Quantitative analysis of mucin and lectin in maxillary sinus fluids in patients with acute and chronic sinusitis. Laryngoscope. 2001;111:240–5. 11210868 10.1097/00005537-200102000-00010

[18] Lund VJ. Nasal physiology: neurochemical receptors, nasal cycle, and ciliary action. Allergy asthma Proc. 1996;17:179–84. 8871735 10.2500/108854196778996877

[19] Dunstan DE, Hamilton-Brown P, Asimakis P, Ducker W, Bertolini J. Shear flow promotes amyloid-beta fibrilization. Protein Eng. Des. Sel. 2009;22:741–6. 19850675 10.1093/protein/gzp059

[20] Wierenga PA, Egmond MR, Voragen AG, de Jongh HH. The adsorption and unfolding kinetics determines the folding state of proteins at the air-water interface and thereby the equation of state. J. Colloid Interface Sci. 2006;299:850–7. 16600281 10.1016/j.jcis.2006.03.016

[21] England RJ, Homer JJ, Knight LC,Ell SR.Nasal pH measurement: a reliable and repeatable parameter. Clin. Otolaryngol. Allied Sci. 1999;24:67–8. 10196653 10.1046/j.1365-2273.1999.00223.x

[22] Braunreuther M, Arenhoevel J, Bej R, Moose C, Mall MA, Haag R, et al. Magnetic microwire rheometer reveals differences in hydrogel degradation via disulfide reducing agents. Soft Matter. 2025;21:427–34. 39704007 10.1039/d4sm01118j

[23] Growdon ME, Schultz AP, Dagley AS, Amariglio RE, Hedden T, Rentz DM, et al. Odor identification and Alzheimer disease biomarkers in clinically normal elderly. Neurology. 2015;84:2153–60. 25934852 10.1212/WNL.0000000000001614 PMC4451046

[24] Van Regemorter V, Hummel T, Rosenzweig F, Mouraux A, Rombaux P, Huart C. Mechanisms Linking Olfactory Impairment and Risk of Mortality. Front. Neurosci. 2020;14:140. 32153360 10.3389/fnins.2020.00140 PMC7046549

[25] Li X, Chen Y, Yang Z, Zhang S, Wei G, Zhang L. Structural insights into the co-aggregation of Abeta and tau amyloid core peptides: Revealing potential pathological heterooligomers by simulations. Int J. Biol. Macromol. 2024;254:127841. 37924907 10.1016/j.ijbiomac.2023.127841

[26] Huang F, Liu Y, Wang Y, Xu J, Lian J, Zou Y, et al. Co-aggregation of alpha-synuclein with amyloid-beta stabilizes beta-sheet-rich oligomers and enhances the formation of beta-barrels. Phys. Chem. Chem. Phys. 2023;25:31604–14. 37964757 10.1039/d3cp04138g PMC10704842

[27] Laos V, Bishop D, Ganguly P, Schonfeld G, Trapp E, Cantrell KL, et al. Catalytic cross talk between key peptide fragments that couple Alzheimer’s disease with amyotrophic lateral sclerosis. J. Am. Chem. Soc. 2021;143:3494–502. 33621087 10.1021/jacs.0c12729

[28] Kawecki GE, King KM, Cramer NA, Bevan DR, Brown AM. Simulations of cross-amyloid aggregation of amyloid-beta and islet amyloid polypeptide fragments. Biophys. J. 2022;121:2002–13. 35538665 10.1016/j.bpj.2022.05.007 PMC9247468

[29] Ge X, Yang Y, Sun Y, Cao W, Ding F. Islet Amyloid Polypeptide Promotes Amyloid-Beta Aggregation by Binding-Induced Helix-Unfolding of the Amyloidogenic Core. ACS Chem. Neurosci. 2018;9:967–75. 29378116 10.1021/acschemneuro.7b00396 PMC5955824

[30] Arotcarena ML, Dovero S, Prigent A, Bourdenx M, Camus S, Porras G, et al. Bidirectional gut-to-brain and brain-to-gut propagation of synucleinopathy in non-human primates. Brain J. Neurol. 2020;143:1462–75. 10.1093/brain/awaa096 32380543

[31] Kim H, Kang SJ, Jo YM, Park S, Yun SP, Lee YS, et al. Novel nasal epithelial cell markers of Parkinson’s disease identified using cells treated with alpha-Synuclein preformed fibrils. J. Clin. Med. 2020;9:2128. 10.3390/jcm9072128 PMC7408990 32640699

[32] Koskinen K, Reichert JL, Hoier S, Schachenreiter J, Duller S, Moissl-Eichinger C, et al. The nasal microbiome mirrors and potentially shapes olfactory function. Sci. Rep. 2018;8:1296. 29358754 10.1038/s41598-018-19438-3 PMC5778015

[33] Gondelaud F, Lozach PY, Longhi S. Viral amyloids: New opportunities for antiviral therapeutic strategies. Curr. Opin. Struct. Biol. 2023;83:102706. 37783197 10.1016/j.sbi.2023.102706

[34] Sonderby TV, Najarzadeh Z, Otzen DE. Functional bacterial amyloids: understanding fibrillation, regulating biofilm fibril formation and organizing surface assemblies. Molecules. 2022;27:4080. 10.3390/molecules27134080 PMC9268375 35807329

[35] Hammarstrom P, Nystrom S. Viruses and amyloids - a vicious liaison. Prion. 2023;17:82–104. 36998202 10.1080/19336896.2023.2194212 PMC10072076

[36] Kell DB, Laubscher GJ, Pretorius E. A central role for amyloid fibrin microclots in long COVID/PASC: origins and therapeutic implications. Biochem. J. 2022;479:537–59. 35195253 10.1042/BCJ20220016 PMC8883497

[37] Ezzat K, Pernemalm M, Palsson S, Roberts TC, Jarver P, Dondalska A, et al. The viral protein corona directs viral pathogenesis and amyloid aggregation. Nat. Commun. 2019;10:2331. 31133680 10.1038/s41467-019-10192-2 PMC6536551

[38] Levine KS, Leonard HL, Blauwendraat C, Iwaki H, Johnson N, Bandres-Ciga S, et al. Virus exposure and neurodegenerative disease risk across national biobanks. Neuron. 2023;111:1086–93.e2. 36669485 10.1016/j.neuron.2022.12.029 PMC10079561

[39] Sampson TR, Challis C, Jain N, Moiseyenko A, Ladinsky MS, Shastri GG, et al. A gut bacterial amyloid promotes alpha-synuclein aggregation and motor impairment in mice. eLife. 2020;9:e53111. 10.7554/eLife.53111 PMC7012599 32043464

[40] Onisiforou A, Charalambous EG, Zanos P. Shattering the amyloid illusion: the microbial enigma of Alzheimer’s disease pathogenesis-from gut microbiota and viruses to brain biofilms. Microorganisms. 2025;13:90. 10.3390/microorganisms13010090 PMC11767882 39858858

[41] Wang J, Gu BJ, Masters CL, Wang YJ. A systemic view of Alzheimer disease - insights from amyloid-beta metabolism beyond the brain. Nat. Rev. Neurol. 2017;13:612–23. 28960209 10.1038/nrneurol.2017.111

[42] Wittung-Stafshede P. Gut power: Modulation of human amyloid formation by amyloidogenic proteins in the gastrointestinal tract. Curr. Opin. Struct. Biol. 2022;72:33–8. 34450484 10.1016/j.sbi.2021.07.009

[43] Grolli S, Merli E, Conti V, Scaltriti E, Ramoni R. Odorant binding protein has the biochemical properties of a scavenger for 4-hydroxy-2-nonenal in mammalian nasal mucosa. FEBS J. 2006;273:5131–42. 17042783 10.1111/j.1742-4658.2006.05510.x

